# Supplementary material for: Phosphorylation of PFKL regulates metabolic reprogramming in macrophages following pattern recognition receptor activation
Source: Nat Commun. 2024 Jul 31;15:6438. doi: 10.1038/s41467-024-50104-7 (PMC11291651; doi:10.1038/s41467-024-50104-7)
Supplement: Supplementary file 1 — Supplementary Information [file 41467_2024_50104_MOESM1_ESM.pdf]

# **Phosphorylation of PFKL regulates metabolic reprogramming in macrophages following pattern recognition receptor activation**

Meiyue Wang[1], Heinrich Flaswinkel[2], Abhinav Joshi[3], Matteo Napoli[4], Sergi Masgrau Alsina[4], Julia M. Kamper[1], Antonia Henne[5], Alexander Heinz[5], Marleen Berouti[1], Niklas A. Schmacke[1], Karsten Hiller[5], Elisabeth Kremmer[2], Benedikt Wefers[6,7], Wolfgang Wurst[6,7], Markus Sperandio[4], Jürgen Ruland[3], Thomas Fröhlich[1], Veit Hornung[1,*]

Affiliations

[1] Gene Center and Department of Biochemistry, Ludwig-Maximilians-Universität München, Munich, Germany

[2] Faculty of Biology, Human Biology and BioImaging, Ludwig-Maximilians-Universität München, Planegg-Martinsried, Germany

[3] Center of Translational Cancer Research, Technische Universität München, Munich, Germany

[4] Faculty of Medicine Biomedical Center, Cardiovascular Physiology and Pathophysiology, Ludwig-Maximilians-Universität München, Planegg-Martinsried, Germany

[5] Institute for Biochemistry, Biotechnology and Bioinformatics, Technische Universität Braunschweig, Braunschweig, Germany

[6]Institute of Developmental Genetics, Helmholtz Zentrum München, Neuherberg, Germany; TUM School of Life Sciences, Technische Universität München, Freising-Weihenstephan, Germany

[7]Deutsches Zentrum für Neurodegenerative Erkrankungen (DZNE) Site Munich, Germany; Munich Cluster for Systems Neurology (SyNergy), Munich, Germany

[*] Correspondence: Veit Hornung (hornung@genzentrum.lmu.de)

## Supplementary Figures

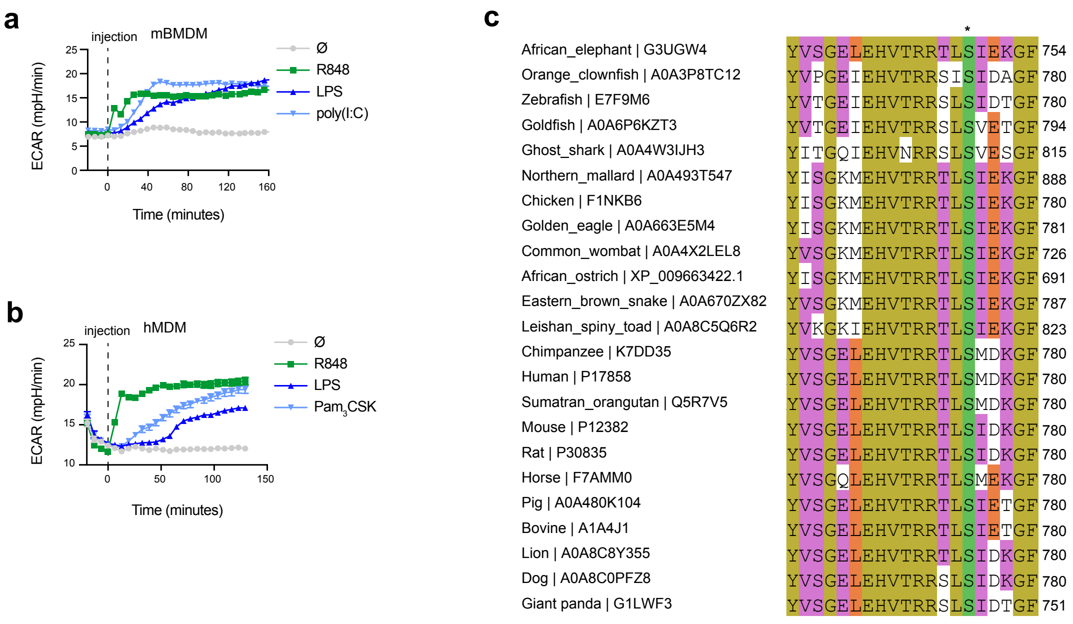


**Supplementary Fig. 1 The Ser775 residue within PFKL is highly conserved across vertebrates.**

**a** and **b**, ECAR was measured over time in response to the indicated TLR agonists in mBMDM (**a**) and hMDM (**b**). Measurement of one representative experiment of two independent experiments is shown. Data are depicted as mean ± SEM (**a**, $n$ = 7; **b**, $n$ = 6 technical replicates). **c**, Multiple sequence alignment of PFKL from different species based on the provided Uniprot entries. Only residues from 761 to 780 (human numbering) are shown. Ser775 (human numbering) is highlighted in green and annotated with an asterisk.

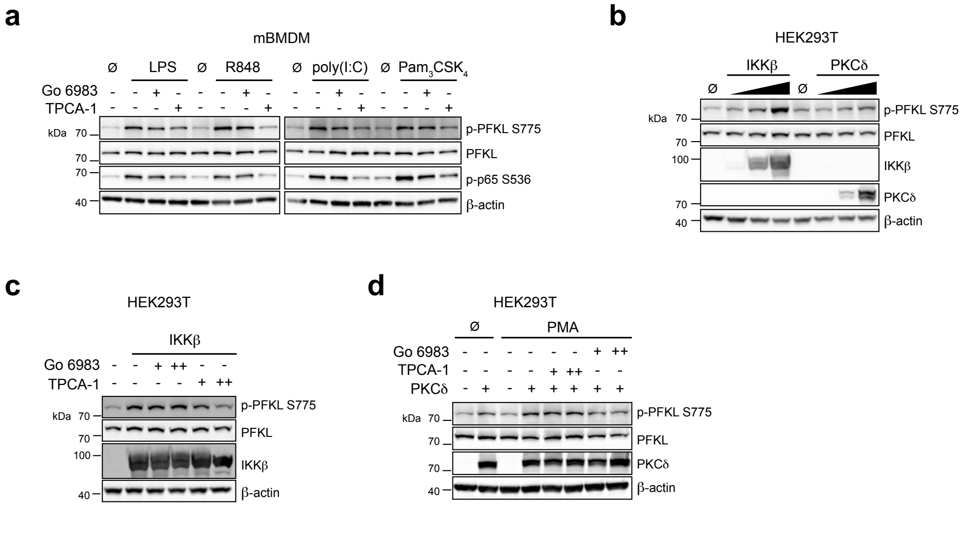


**Supplementary Fig. 2 IKKβ and PKCδ overexpression leads to PFKL Ser775 phosphorylation.**

Immunoblot analysis of indicated proteins. **a**, mBMDM were pretreated with DMSO, TPCA-1 (5 μM), or Go 6983 (5 μM) for 1 h and then stimulated with the indicated TLR agonists for 1 h (LPS, poly(I:C) and $Pam_3CSK_4$) or for 30 min (R848). **b**, HEK293T cells were transfected with IKKβ or PKCδ (0, 0.25, 0.5 and 1 μg) for 24 h. **c**, HEK293T cells were transfected with IKKβ (1 μg). 16 h post transfection, the indicated inhibitors (+, 1 μM; ++, 5 μM) were added for 6 h. **d**, HEK293T cells were transfected with PKCδ (1 μg). 16 h post transfection, cells were treated with the indicated inhibitors (+, 1 μM; ++, 5 μM) for 5 h, followed by PMA (10 ng/ml) stimulation for 1 h. β-actin was used as a loading control. **a-d** are representative of three independent experiments.

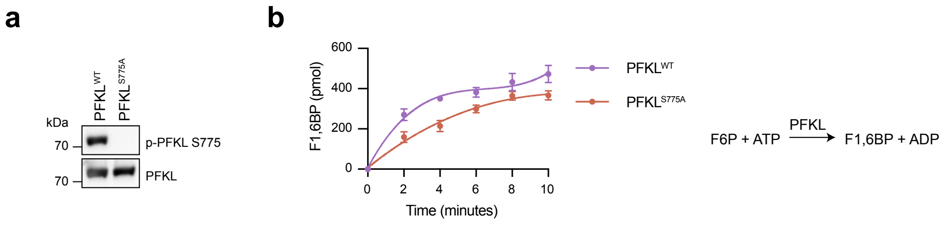


**Supplementary Fig. 3 PFKL$^{WT}$ is more active than PFKL$^{S775A}$.**

**a**, Immunoblot analysis of purified PFKL$^{WT}$ and PFKL$^{S775A}$. One representative of three independent experiments is shown. **b**, F1,6BP formation from in vitro enzymatic assays using purified PFKL$^{WT}$ and PFKL$^{S775A}$ was measured by LC-MS. Data are presented as mean ± SEM (*n* = 3). The interpolated line is derived from fitting the data with a third-order polynomial model.

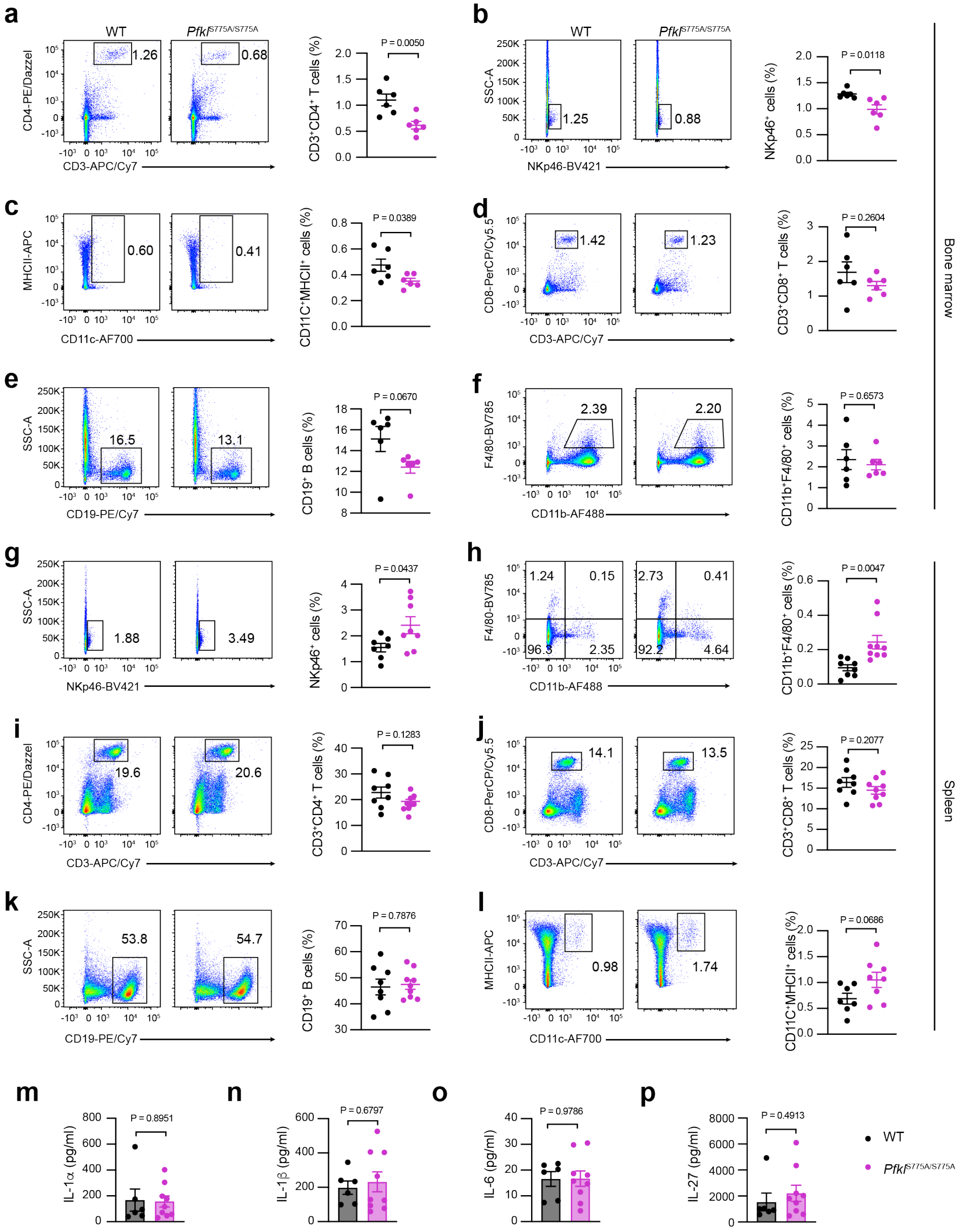


**Supplementary Fig. 4 Characterization of *Pfkl*$^{S775A/S775A}$ mice.**

**a-f**, Bone marrow cells were isolated from WT and *Pfkl*$^{S775A/S775A}$ mice and analyzed by flow cytometry for the indicated cell surface markers. Gating strategies for each immune cell type are shown, and the numbers next to the outlined areas indicate the percentage of each immune cell type among living cells (left panel). The percentages of indicated immune cells among living cells

were summarized (right panel). Data are presented as mean ± SEM (*n* = 6), statistics indicate unpaired two-tailed student's *t*-test. **g-l**, Splenocytes were isolated from WT and *Pfkl*$^{S775A/S775A}$ mice and analyzed by flow cytometry for the indicated cell surface markers. Gating strategies for each immune cell type were shown, and the numbers next to the outlined areas indicate the percentage of each immune cell type among living cells (left panel). The percentages of indicated immune cells among living cells were summarized (right panel). Data are presented as mean ± SEM (*n* = 7-9 as indicated), statistics indicate unpaired two-tailed student's *t*-test. **m-p**, IL-1α (**m**), IL-1β (**n**), IL-6 (**o**), and IL-27 (**p**) in the serum from WT and *Pfkl*$^{S775A/S775A}$ mice were measured by flow cytometry. Data are presented as mean ± SEM (WT mice; *n* = 6; *Pfkl*$^{S775A/S775A}$ mice, *n* = 9), statistics indicate unpaired two-tailed student's *t*-test.

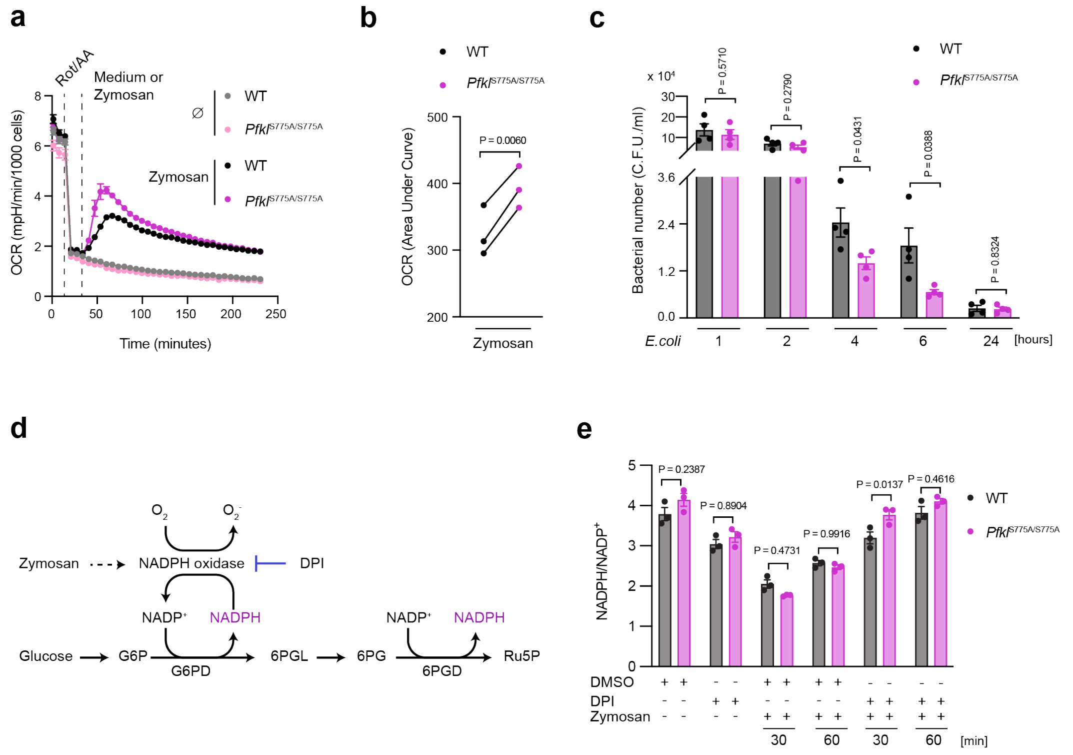


**Supplementary Fig. 5 *Pfkl*$^{S775A/S775A}$ macrophages generate more ROS and kill bacteria more effectively**.

mBMDM were primed with 20 ng/ml mouse IFNγ overnight before stimulation or infection. **a**, OCR was measured over time in response to the indicated compounds and zymosan (100 µg/ml) in WT and *Pfkl*$^{S775A/S775A}$ mBMDM. Rot/AA, rotenone and antimycin A (0.5 µM). Measurement of one representative experiment is shown. Data are presented as mean ± SEM (∅, *n* = 5; zymosan, *n* = 6 technical replicates). **b**, Oxygen consumption after zymosan injection from a was represented by calculating the area under the curve of the complete kinetic range between activation and return to basal rates. Data are presented as mean ± SEM (*n* = 3), statistics indicate paired two-tailed student's *t*-test. **c**, WT and *Pfkl*$^{S775A/S775A}$ mBMDM were infected with *E. coli* at a multiplicity of infection of 10 and colony-forming units (C.F.U.) recovered after bacterial phagocytosis for the indicated time were quantified and are presented as mean ± SEM (*n* = 4), statistics indicate unpaired two-tailed student's *t*-test. **d**, Model of ROS production under NADPH regulation. **e**, The ration of NADPH/NADP$^{+}$ under the indicated conditions. Data are presented as mean ± SEM (*n* = 3), statistics indicate two-way ANOVA with Šidák's correction for multiple comparisons test.

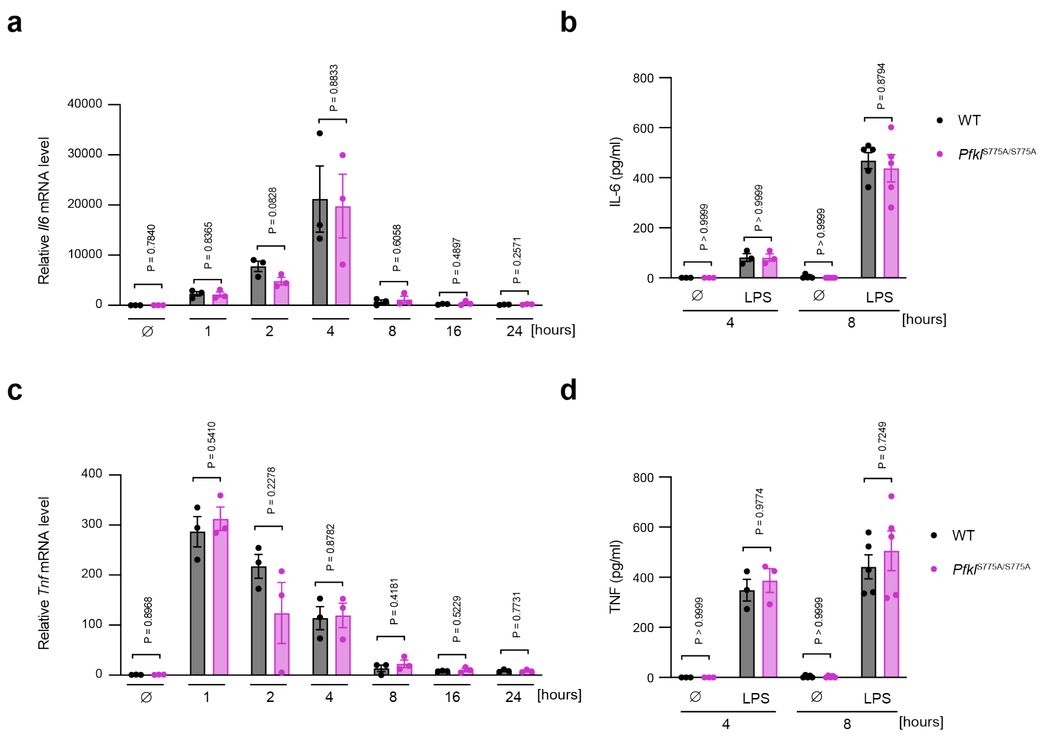


**Supplementary Fig. 6 PFKL Ser775 phosphorylation is dispensable for LPS-induced IL-6 and TNF production.**

**a**, LPS-induced *Il6* mRNA levels in WT and *Pfkl*$^{S775A/S775A}$ mBMDM were measured by qPCR and normalized to *Actb* mRNA levels. Data are presented as mean ± SEM ($n$ = 3), statistics indicate unpaired two-tailed student's *t*-test. **b**, LPS-induced IL-6 production in WT and *Pfkl*$^{S775A/S775A}$ mBMDM was measured by ELISA. Data are presented as mean ± SEM (4 h, $n$ = 3; 8 h, $n$ = 5), statistics indicate two-way ANOVA with Šidák's correction for multiple comparisons test. **c**, LPS-induced *Tnf* mRNA levels in WT and *Pfkl*$^{S775A/S775A}$ mBMDM were measured by qPCR and normalized to *Actb* mRNA levels. Data are presented as mean ± SEM ($n$ = 3), statistics indicate unpaired two-tailed student's *t*-test. **d**, LPS-induced TNF production in WT and *Pfkl*$^{S775A/S775A}$ mBMDM was measured by ELISA. Data are presented as mean ± SEM (4 h, $n$ = 3; 8 h, $n$ = 5), statistics indicate two-way ANOVA with Šidák's correction for multiple comparisons test.

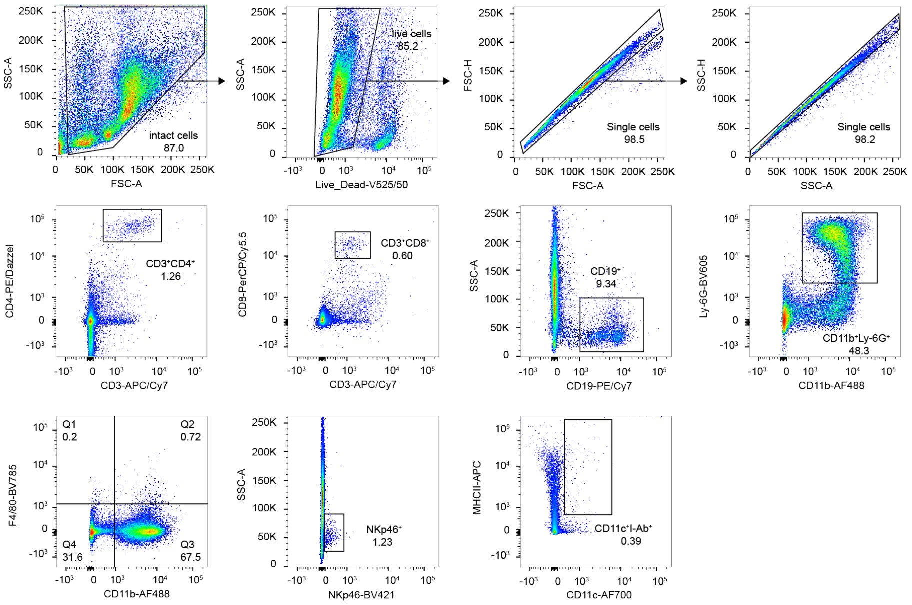


**Supplementary Fig. 7 Gating strategy for the identification of immune cell subsets in bone marrow.**

An exemplary gating strategy for the identification of different immune cell subsets in bone marrow is illustrated. The top row demonstrates the consecutive gating strategy utilized to identify single cells. Exemplary percentages for specific immune cell subsets are provided, based on data from one representative experiment.

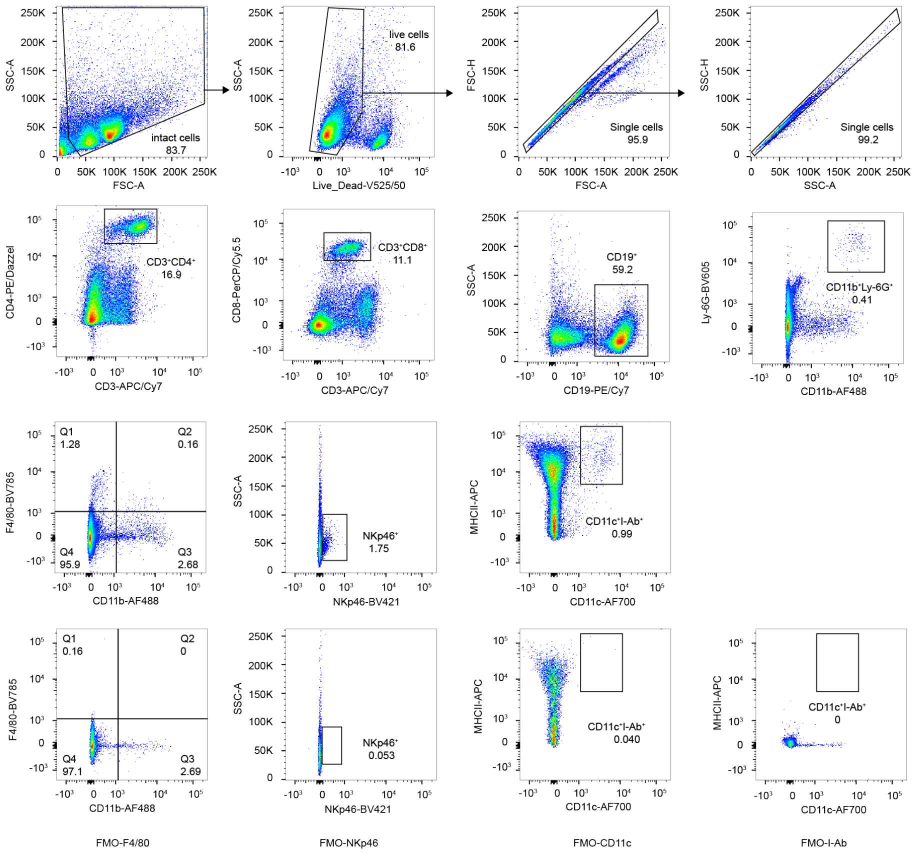


**Supplementary Fig. 8 Gating strategy for the identification of immune cell subsets in spleen.** An exemplary gating strategy for the identification of different immune cell subsets in spleen is illustrated. The top row demonstrates the consecutive gating strategy utilized to identify single cells. Exemplary percentages for specific immune cell subsets are provided, based on data from one representative experiment.

# Supplementary Source Data

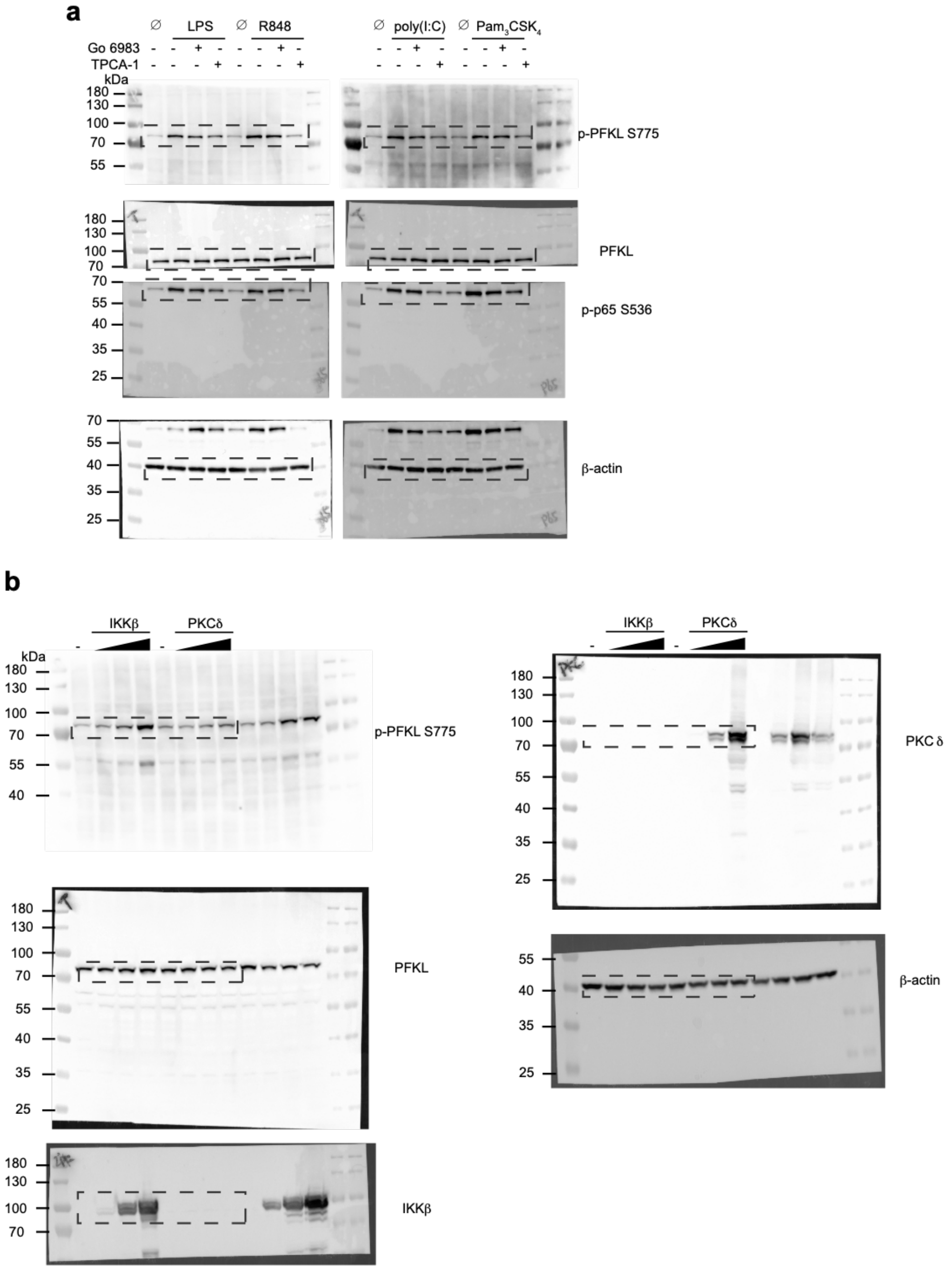


# Source data of Supplementary Fig. 2a and 2b

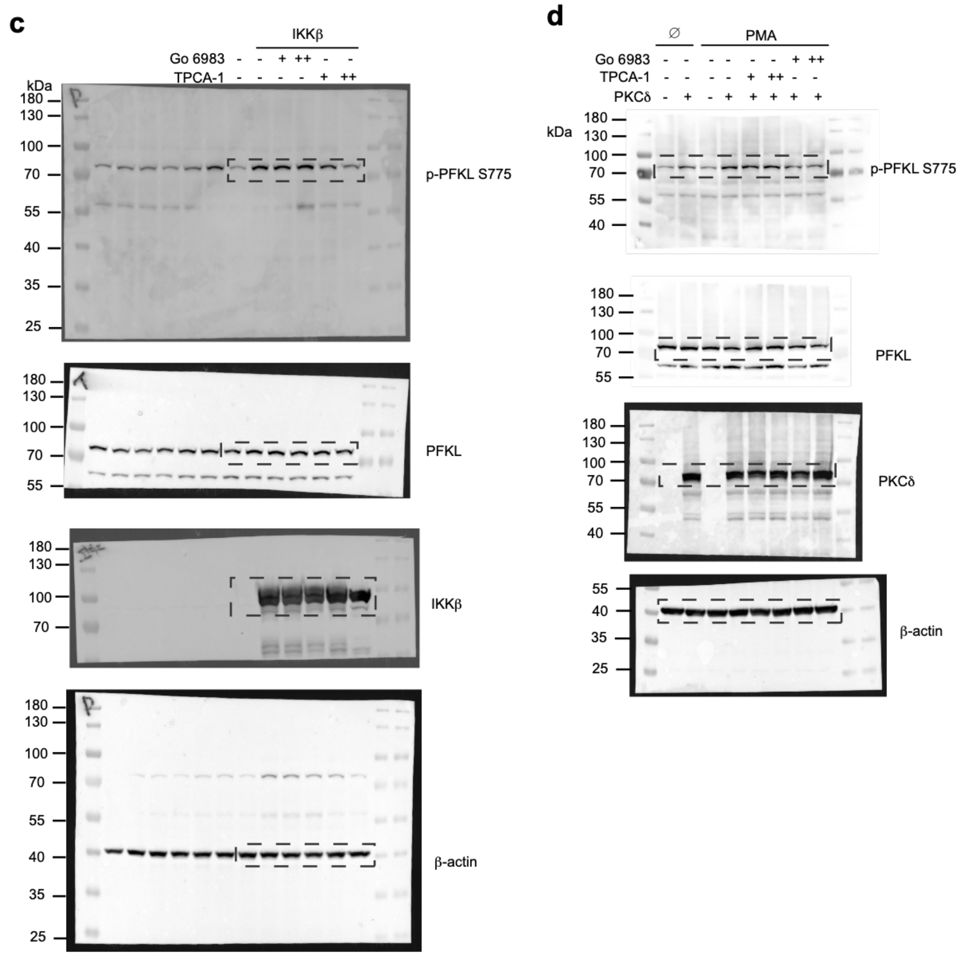


**Source data of Supplementary Fig. 2c and 2d**

**a**

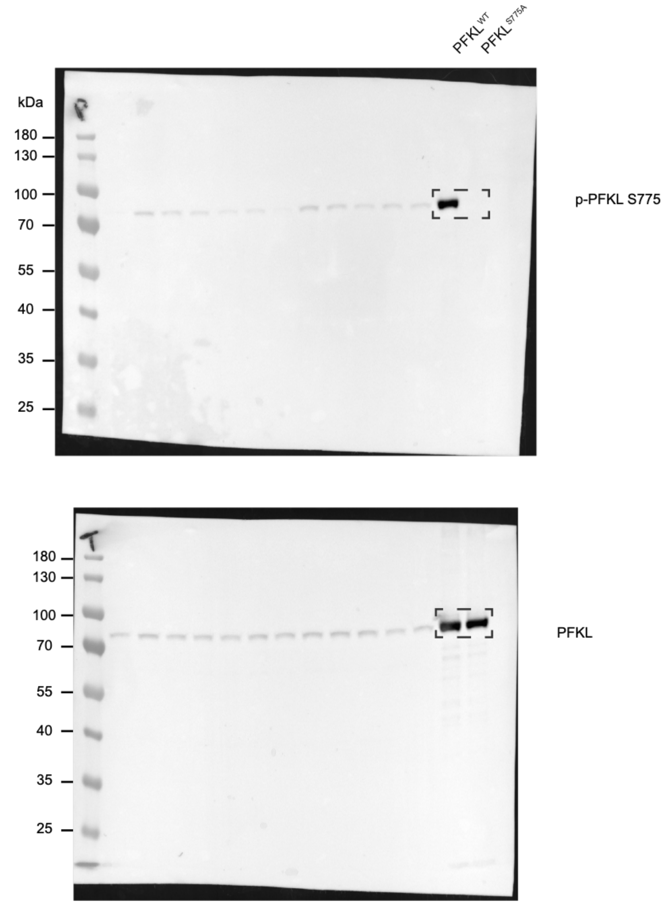


**Source data of Supplementary Fig. 3a**
