# Supplementary material for: Phosphorylation of PFKL regulates metabolic reprogramming in macrophages following pattern recognition receptor activation
Source: Nat Commun. 2024 Jul 31;15:6438. doi: 10.1038/s41467-024-50104-7 (PMC11291651; doi:10.1038/s41467-024-50104-7)
Supplement: Supplementary file 3 — Description of Additional Supplementary Files [file 41467_2024_50104_MOESM3_ESM.pdf]

Description of Additional Supplementary Files

File Name: Supplementary Data 1
Description: The table displays the predicted kinase phosphorylation scores for the S775 residue of mouse and human PFKL as provided by https://www.phosphosite.org/, which is based on Johnson et al. “An atlas of substrate specificities for the human serine/threonine kinome” Nature 2023 Jan 11; 613, 759–766; DOI: 10.1038/s41586-022-05575-3.
