# Supplementary material for: Phosphorylation of PFKL regulates metabolic reprogramming in macrophages following pattern recognition receptor activation
Source: Nat Commun. 2024 Jul 31;15:6438. doi: 10.1038/s41467-024-50104-7 (PMC11291651; doi:10.1038/s41467-024-50104-7)
Supplement: Supplementary file 5 — Reporting Summary [file 41467_2024_50104_MOESM5_ESM.pdf]

nature portfolio

Corresponding author(s): Veit Hornung

Last updated by author(s): Jun 10, 2024

Reporting Summary

Nature Portfolio wishes to improve the reproducibility of the work that we publish. This form provides structure for consistency and transparency in reporting. For further information on Nature Portfolio policies, see our Editorial Policies and the Editorial Policy Checklist.

Statistics

For all statistical analyses, confirm that the following items are present in the figure legend, table legend, main text, or Methods section.

n/a	Confirmed	
☐	☒	The exact sample size (*n*) for each experimental group/condition, given as a discrete number and unit of measurement
☐	☒	A statement on whether measurements were taken from distinct samples or whether the same sample was measured repeatedly
☐	☒	The statistical test(s) used AND whether they are one- or two-sided<br>*Only common tests should be described solely by name; describe more complex techniques in the Methods section.*
☒	☐	A description of all covariates tested
☐	☒	A description of any assumptions or corrections, such as tests of normality and adjustment for multiple comparisons
☐	☒	A full description of the statistical parameters including central tendency (e.g. means) or other basic estimates (e.g. regression coefficient) AND variation (e.g. standard deviation) or associated estimates of uncertainty (e.g. confidence intervals)
☐	☒	For null hypothesis testing, the test statistic (e.g. *F*, *t*, *r*) with confidence intervals, effect sizes, degrees of freedom and *P* value noted<br>*Give P values as exact values whenever suitable.*
☒	☐	For Bayesian analysis, information on the choice of priors and Markov chain Monte Carlo settings
☒	☐	For hierarchical and complex designs, identification of the appropriate level for tests and full reporting of outcomes
☒	☐	Estimates of effect sizes (e.g. Cohen's *d*, Pearson's *r*), indicating how they were calculated

Our web collection on statistics for biologists contains articles on many of the points above.

Software and code

Policy information about availability of computer code

Data collection	The following software was used for data collection: BD FACSDiva 8.0; Seahorse Wave Analyzer software; Bio-Rad CFX Manager 3.1; Gen5 1.11
Data analysis	All statistical analyses were performed with GraphPad Prism 9.<br>Flow cytometry data were analysed using FlowJo 10.5.3.<br>Mass spectrometry raw files were processed using Analyst and Peak View V2.2 software.<br>WB quantification was performed using ImageJ and Image Lab.

For manuscripts utilizing custom algorithms or software that are central to the research but not yet described in published literature, software must be made available to editors and reviewers. We strongly encourage code deposition in a community repository (e.g. GitHub). See the Nature Portfolio guidelines for submitting code & software for further information.

Data

Policy information about availability of data

All manuscripts must include a data availability statement. This statement should provide the following information, where applicable:

- Accession codes, unique identifiers, or web links for publicly available datasets
- A description of any restrictions on data availability
- For clinical datasets or third party data, please ensure that the statement adheres to our policy

Accession codes for PFKL sequences (Supplementary Fig. 1c) are provided. Predicted kinases for directly phosphorylating human and mouse PFKL are provided in

Supplementary Data. Uncropped immunoblots for Fig. 1-3 and 6 are provided in the Source Data file. Uncropped immunoblots for Supplementary Fig. 2 and 3 are provided in the Supplementary Information file. Materials and reagents are available from the corresponding author upon request.

Research involving human participants, their data, or biological material

Policy information about studies with human participants or human data. See also policy information about sex, gender (identity/presentation), and sexual orientation and race, ethnicity and racism.

Reporting on sex and gender	PBMCs were isolated from the leukocyte reduction system chambers left over from platelet donation from healthy donors. Neither information on sex, gender, race, ethnicity are available to us.
Reporting on race, ethnicity, or other socially relevant groupings	see above
Population characteristics	see above
Recruitment	see above
Ethics oversight	Approval from the relevant ethics committee and informed consent from all donors according to the Declaration of Helsinki were obtained (project number: 19-238, Ethics Committee of the Medical Faculty of Ludwig-Maximilians-University Munich).

Note that full information on the approval of the study protocol must also be provided in the manuscript.

Field-specific reporting

Please select the one below that is the best fit for your research. If you are not sure, read the appropriate sections before making your selection.

☒ Life sciences ☐ Behavioural & social sciences ☐ Ecological, evolutionary & environmental sciences

For a reference copy of the document with all sections, see nature.com/documents/nr-reporting-summary-flat.pdf

Life sciences study design

All studies must disclose on these points even when the disclosure is negative.

Sample size	No statistical methods were used to predetermine sample size, sample size were chosen based on what is common practice in the field.
Data exclusions	A ROUT outlier correction was conducted for the in vivo cytokine measurements (Fig. 4i-j).
Replication	All experiments were independently repeated at least twice, as indicated in the figure legends.
Randomization	Randomization was not performed for this study, as it was unnecessary to control for factors that would not be under direct experimental control.
Blinding	Blinding was not required as data were based on quantitative analysis phenotypes and no subjective analysis were performed.

Reporting for specific materials, systems and methods

We require information from authors about some types of materials, experimental systems and methods used in many studies. Here, indicate whether each material, system or method listed is relevant to your study. If you are not sure if a list item applies to your research, read the appropriate section before selecting a response.

Materials & experimental systems

n/a	Involved in the study	
☐	☒	Antibodies
☐	☒	Eukaryotic cell lines
☒	☐	Palaeontology and archaeology
☐	☒	Animals and other organisms
☒	☐	Clinical data
☒	☐	Dual use research of concern
☒	☐	Plants

Methods

n/a	Involved in the study	
☒	☐	ChIP-seq
☐	☒	Flow cytometry
☒	☐	MRI-based neuroimaging

Antibodies

Antibodies used	Immunoblot:<br>pSer775-PFKL (1:100; in house)<br>PFKL (1:250; in house)<br>beta-actin-HRP (1:5000; Santa Cruz sc-47778)<br>HI1F1a (D1S7W; 1:1000; Cell signaling 36169S)<br>mouse IL-1beta (1:1000; R&D system AF-401-NA)<br>IgG (H+L) Donkey anti-goat, HRP (1:3000; Invitrogen A15999)<br>anti-rabbit IgG-HRP (1:3000; Cell signaling 7074)<br>p-NF-KB p65 (Ser536) (93H1; 1:1000; Cell signaling 3033)<br>HA Tag (6E2) mouse mAb, HRP conjugate (1:000, Cell signaling 2999S)<br>HRP-goat anti-mouse IgG, Fcgamma secondary (1:3000; Jackson ImmunoResearch 115-035-071)<br>IKKb (D30C6) rabbit (1:1000; Cell signaling 8943S)<br>p-AKT T308 rabbit (C31E5E; 1:500; Cell signaling 13038S)<br>p-AKT S473 rabbit (D9E; 1:1000; Cell signaling 4060S)<br>AKT (pan) rabbit (C67E7; 1:1000; Cell signaling 4691S)<br><br>Flow cytometry:<br>APC/Cyanine7 anti-mouse CD3 (145-2C11; 1:50; BioLegend 100330)<br>PE/Dazzle 594 anti-mouse CD4 (GK 1.5; 1:100; BioLegend 100456)<br>PerCP/Cyanine5.5 anti-mouse CD8b.2 (53-5.8; 1:100; BioLegend 140418)<br>PE/Cyanine7 anti-mouse CD19 (6D5; 1:200; BioLegend 115520)<br>Brilliant Violet 421 anti-mouse CD335 (NKp46) (29A1.4; 1:50; BioLegend 137612)<br>Alexa Fluor 488 anti-mouse/human CD11b (M1/70; 1:200; BioLegend 101217)<br>Brilliant Violet 785 anti-mouse F4/80 (BM8; 1:500; BioLegend 123141)<br>Alexa Fluor 700 anti-mouse CD11c (N418; 1:200; BioLegend 117320)<br>APC anti-mouse I-Ab (AF6-120.1; 1:50; BioLegend 116418)<br>Brilliant Violet 605 anti-mouse Ly-6G (1A8; 1:200; BioLegend 127639)
Validation	Except for the anti-pSer775 PFKL and anti-PFKL antibodies, we did not validate the antibodies ourselves. Appropriate controls were performed to ensure that appropriate conclusions were drawn. Validation data of all commercial antibodies are available on vendor websites.

Eukaryotic cell lines

Policy information about cell lines and Sex and Gender in Research

Cell line source(s)	HEK293T cells were from DSMZ (ACC 635).
Authentication	HEK293T cells were not additionally authenticated.
Mycoplasma contamination	HEK293T cells were tested negative for mycoplasma contamination at the beginning of the study, but was not tested routinely thereafter.
Commonly misidentified lines<br>(See ICLAC register)	No commonly misidentified cell lines were used in this study.

Animals and other research organisms

Policy information about studies involving animals; ARRIVE guidelines recommended for reporting animal research, and Sex and Gender in Research

Laboratory animals	Male or female WT and PfklS775A/S775A mice were used for BMDM isolation (please see methods section for details).<br>All mice were housed in standard cages in a specific pathogen-free facility (21 ± 1°C, on a 12-h light/dark cycle, with average humidity of about 55%) with ad libitum access to food and water in the animal facility at the Center for Neuropathology.
Wild animals	No wild animals were used in this study.
Reporting on sex	To avoid any influence of the estrous cycle, all experiments concerning metabolomic readouts were performed in male mice.
Field-collected samples	This study did not involve any field-collected samples.
Ethics oversight	All mice were handled according to institutional guidelines approved by the animal welfare and use committee of the goverment of Upper Bavaria.

Note that full information on the approval of the study protocol must also be provided in the manuscript.

Plants

Seed stocks	N/A
Novel plant genotypes	N/A
Authentication	N/A

Flow Cytometry

Plots

Confirm that:

☒ The axis labels state the marker and fluorochrome used (e.g. CD4-FITC).

☒ The axis scales are clearly visible. Include numbers along axes only for bottom left plot of group (a 'group' is an analysis of identical markers).

☒ All plots are contour plots with outliers or pseudocolor plots.

☒ A numerical value for number of cells or percentage (with statistics) is provided.

Methodology

Sample preparation	Bone marrow cells from WT and PfklS775A/S775A mice were flushed from the femur and tibia. Splenocytes were obtained by thoroughly mincing the spleen with scissors. Clumps and debris in the cell suspensions were removed by passing the cell suspension twice through a 100 um cell sieve. RBC lysis buffer was used to remove red blood cells. Cells were incubated with FcR blocking reagent for 20-30 min at 4°C before incubation with antibodies.
Instrument	Cells were analyzed on a BD LSR Fortessa.
Software	BD FACSDiva 8.0.2 was used for data collection.<br>Flow cytometry data were analyzed using FlowJo 10.5.3.
Cell population abundance	Conclusions on cell population abundance were made when analysing the percentages of each type of immune cell in the bone marrow and spleen of mice. For these studies, the gate was drawn based on the FMO controls and then applied to all other samples in the respective dataset.
Gating strategy	Cells were first gated on FSC-A vs SSC-A to exclude debris. Live cells were identified by gating on Live/Dead Aqua staining. FSC-A vs FSC-H as well as SSC-A vs SSC-H were followed to discriminate single cells from doublets. Each population of immune cells was then gated based on FMO control.

☒ Tick this box to confirm that a figure exemplifying the gating strategy is provided in the Supplementary Information.
